# Brain-encysting trematodes (*Euhaplorchis californiensis*) decrease raphe serotonergic activity in California killifish (*Fundulus parvipinnis*)

**DOI:** 10.1242/bio.049551

**Published:** 2020-07-08

**Authors:** Siri H. Helland-Riise, Marco A. Vindas, Ida B. Johansen, Lauren E. Nadler, Kelly L. Weinersmith, Ryan F. Hechinger, Øyvind Øverli

**Affiliations:** 1 Department of Paraclinical Sciences, Faculty of Veterinary Medicine, Norwegian University of Life Sciences, Oslo, Norway 1407; 2 Marine Biology Research Division, Scripps Institution of Oceanography, University of California, San Diego, San Diego, CA 92037, USA; 3 Department of BioSciences, Rice University, Houston, TX 77005, USA

**Keywords:** Parasite density, Parasite intensity, Experimental infections, Neurobiology, Parasite alterations, Serotonin

## Abstract

Modulation of brain serotonin (5-HT) signalling is associated with parasite-induced changes in host behaviour, potentially increasing parasite transmission to predatory final hosts. Such alterations could have substantial impact on host physiology and behaviour, as 5-HT serves multiple roles in neuroendocrine regulation. These effects, however, remain insufficiently understood, as parasites have been associated with both increased and decreased serotonergic activity. Here, we investigated effects of trematode *Euhaplorchis californiensis* metacercariae on post-stress serotonergic activity in the intermediate host California killifish (*Fundulus parvipinnis*). This parasite is associated with conspicuous behaviour and increased predation of killifish by avian end-hosts, as well as inhibition of post-stress raphe 5-HT activity. Until now, laboratory studies have only been able to achieve parasite densities (parasites/unit host body mass) well below those occurring in nature. Using laboratory infections yielding ecologically relevant parasite loads, we show that serotonergic activity indeed decreased with increasing parasite density, an association likely indicating changes in 5-HT neurotransmission while available transmitter stores remain constant. Contrary to most observations in the literature, 5-HT activity increased with body mass in infected fish, indicating that relationships between parasite load and body mass may in many cases be a real underlying factor for physiological correlates of body size. Our results suggest that parasites are capable of influencing brain serotonergic activity, which could have far-reaching effects beyond the neurophysiological parameters investigated here.

## Abstract (precis)

**Summary:** Post-stress 5-HT signalling decrease with parasite density in killifish infected with brain-dwelling *Euhaplorchis californiensis* metacercariae, in a manner consistent with decreased impulse control and increased risk taking in infected fish.

## INTRODUCTION

Many parasites alter their host's phenotype in ways that increase parasite fitness (Holmes et al., 1972; Moore and Gotelli, 1990; Combes, 1991; Poulin, 1994, 2010; Barber et al., 2000; Moore, 2002, 2013; Lefèvre et al., 2009). Remarkable examples of parasite-induced behavioural alterations that ultimately facilitate parasite reproduction include attraction to olfactory cues of feline predators in rats, and water-seeking behaviour in otherwise terrestrial crickets (Berdoy et al., 2000; Thomas et al., 2002). However, the physiological mechanisms driving these behavioural changes associated with parasite infection remain poorly understood.

It has long been debated whether host behavioural changes in response to infection are indirect consequences of parasitism (i.e. pathological by-products) or the result of direct ‘active’ parasite manipulation (e.g. secreting neuroactive substances, reviewed by Adamo, 2002, 2013; Herbison, 2017). For instance, female sticklebacks (*Gasterosteus aculeatus*) infected with larval tapeworms (*Schistocephalus solidus*) show decreased antipredator behaviour and increased brain serotonin (5-Hydroxytryptamine, 5-HT) metabolism (Øverli et al., 2001, Hafer and Milinski, 2016). However, these host phenotypic changes may simply be the result of the infections being quite debilitating (but see Talarico et al., 2017), as the neuroendocrine responses of infected sticklebacks are consistent with chronic stress, i.e. possibly an indirect effect of parasite infection (Winberg and Nilsson, 1993; Øverli et al., 1999).

In other systems, the evidence for direct manipulation is stronger. For example, California killifish (*Fundulus parvipinnis*) infected by the brain-encysting trematode *Euhaplorchis californiensis* are both more vulnerable to predation by avian final hosts (the final host in the life cycle for *E. californiensis*; Lafferty and Morris, 1996) and exhibit a parasite density-dependent decrease in post-stress 5-HT signalling (Shaw et al., 2009). This change in neurochemistry is unlikely to occur as an incidental pathological effect of infection, as chronic stress increases brain serotonergic signalling in other teleost species (Winberg and Nilsson, 1993). Further, this neurochemical profile is consistent with increased risk-taking behaviour during stress (which would likely facilitate predation), as stress induced 5-HT release inhibits activity (e.g. Vindas et al., 2016).

The phylogenetically ancient neurotransmitter/neuromodulator 5-HT serves multiple crucial roles in neuroendocrine regulation. Throughout the vertebrate phylogenetic tree, this monoamine regulates a wide range of central biological processes, including energy regulation, neural plasticity, aggression, behavioural and emotional impulse control, as well as neuroendocrine responses to stress (Lanfumey et al., 2008; Andrews et al., 2015). Moreover, it has been proposed that 5-HT signalling increases in conditions that require the reallocation of energy resources (Andrews et al., 2015). For example, serotonergic activity increases in response to stressful situations, which facilitates reallocation from processes such as growth and reproduction towards cognition and coping behaviours (Andrews et al., 2015). Therefore, if parasites are capable of directly influencing brain serotonergic activity during stress, these effects could have wide ranging impacts on the host's phenotype. Shaw et al. (2009) speculated that *E. californiensis*-infection could disrupt behavioural stress responses (e.g. anti-predator responses) in killifish by interfering with 5-HT signalling post stress. While the results corroborated this hypothesis, the experimental infection protocol yielded parasite densities far lower (two to nine parasites per g body mass; Shaw et al., 2009) than what is observed in wild adult fish (300–900 parasites per g body mass; Shaw and Øverli, 2012). Therefore, investigating whether *E. californiensis* infection supresses post-stress serotonergic activity at ecologically realistic densities is critical to evaluate the possible adaptive nature of infection-induced host behavioural changes.

Moreover, assessing serotonergic signalling requires careful interpretation of observed tissue concentrations of 5-HT, its principle catabolite 5-hydroxyindoleacetic acid (5-HIAA), and the relative ratio between the two. Following release and re-uptake of 5-HT, the monoamine is broken down by the enzyme monoamine oxidase to its catabolite, 5-HIAA. Therefore, concentrations of 5-HIAA are indicative of 5-HT neurotransmission while concentrations of 5-HT indicate available 5-HT synthesis/stores. Thus, as a biochemical proxy of serotonergic activation it is common to report the ratio of the catabolite (5-HIAA) to neurotransmitter (5-HT) concentrations (5-HIAA/5-HT ratio, hereafter referred to as serotonergic activity; Shannon et al., 1986; Winberg and Nilsson, 1993; Fillenz, 1993; Øverli et al., 1999; Summers et al., 2003; Andrews et al., 2015). Strictly, however, complete interpretation of signalling dynamics depends also on knowing individual concentrations of each neurochemical compound. Although previous studies have reported a relationship between *E. californiensis* infection and 5-HIAA/5-HT ratio (Shaw et al., 2009), none have yet specified which compound in the ratio drives the observed effects. Hence, we do not know if *E. californiensis* targets serotonin neurotransmission, synthesis, or both. Potential modification of serotonergic signalling (i.e. release and activation of post-synaptic neurons) is more likely to be able to rapidly affect behaviour than changes in serotonin synthesis. Rather, increased use of the transmitter is under normal circumstances compensated for by increased synthesis, yielding fluctuating catabolite concentrations while 5-HT stores remain rather stable. Further support for the idea that 5-HT stores are tightly regulated is gained from studies manipulating availability of the rate limiting substrate for 5-HT synthesis, the essential amino acid tryptophan (TRP). Increased dietary TRP typically results in increased utilisation (i.e. 5-HIAA concentrations), rather than affecting brain 5-HT stores (Winberg et al., 2001; Martins et al., 2013; Morandini et al., 2019). We therefore predict that *E. californiensis* infection targeting of 5-HT neurotransmission should be reflected in altered 5-HIAA, but not 5-HT, dynamics.

In the context of host phenotype alterations, parasite load has been quantified using both parasite intensity (total number of parasites in an infected host) and parasite density (total number of parasites per g host body mass; Lafferty and Morris, 1996; Shirakashi and Goater, 2001; Shaw et al., 2009; Shaw and Øverli, 2012; Santos and Santos, 2013). Very few studies address host phenotypic changes in relation to both intensity and density (e.g. Weinersmith et al., 2016). For example, if *E*. *californiensis* metacercariae actively influence neurotransmission in brain tissue, we would predict a stronger relationship between serotonergic activity and parasite density (number of parasites per host body mass, which would be correlated strongly with brain mass) than with intensity (number of parasites in a host). Thus, to identify the precise mechanism driving parasite-mediate host phenotypic alternations, experiments should strive to produce ecologically relevant infection levels, and distinguish between infection intensity and density-dependent effects on physiology.

Because it has been speculated that *E. californiensis*-infection disrupts stress responses in killifish by interfering with serotonergic activity (Shaw et al., 2009), this study aimed to investigate post-stress serotonergic activity in response to ecologically relevant *E. californiensis* infection loads. This is the first time that serotonergic neurochemistry has been studied in killifish subjected to repeated experimental infections, mimicking natural infections and yielding parasite densities observed in wild adult fish. To achieve this, we used a recently developed laboratory protocol yielding parasite intensities comparable to those in wild fish (Helland-Riise et al., 2020). To study serotonergic responses, we used a series of sequential stressors: transport stress combined with exposure to a novel environment, a simulated predator attack, and acute confinement in a small volume of water. We hypothesized that serotonergic activity would decrease with increasing parasite density and that this decrease will be reflected in actual changes in 5-HT neurotransmission but not transmitter concentrations.

## RESULTS

**Fig. 1.**
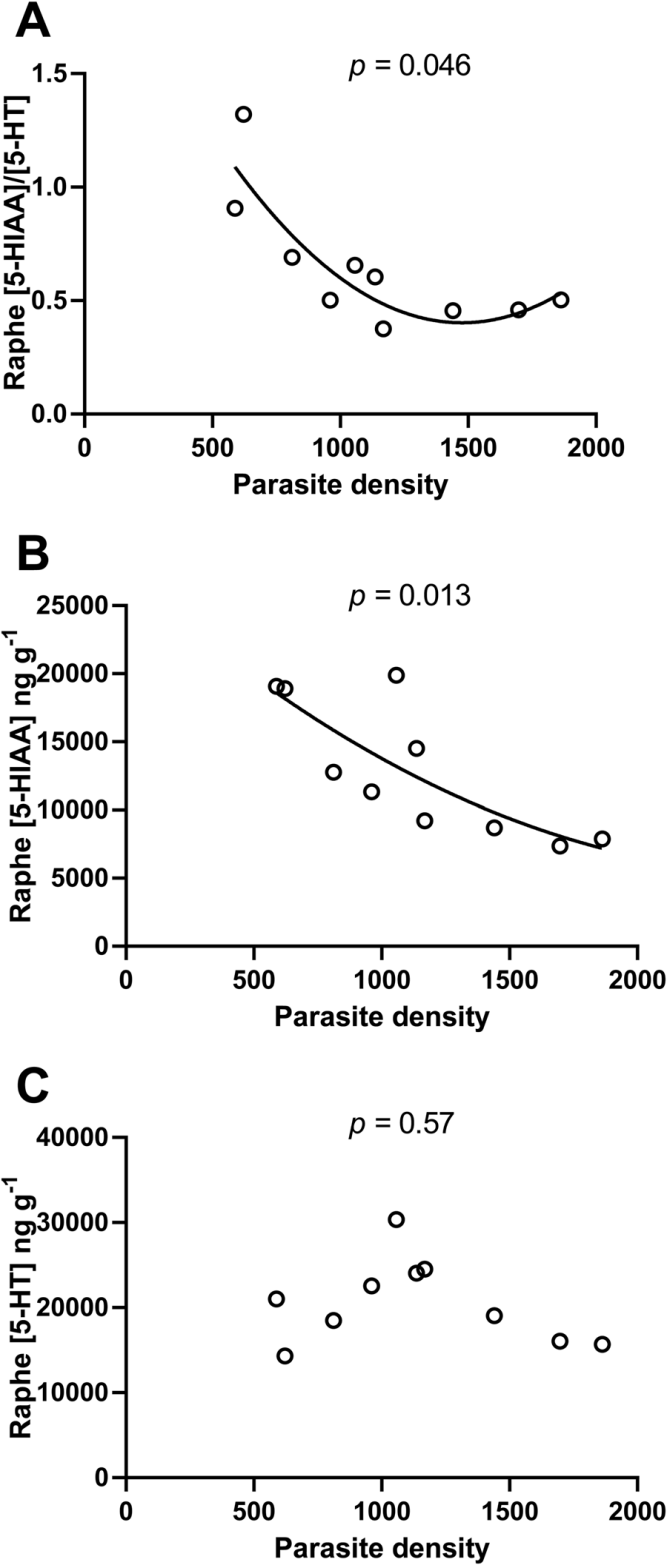
**Relationship between brain density of *E.californiensis* metacercariae and serotonergic neurochemistry in California killifish (*F.parvipinnis*).** (A) Relative concentration ratio of the 5-HT catabolite 5-HIAA to the serotonin neurotransmitter (5-HIAA/5-HT). (B) Concentration of 5-HIAA and (C) concentration of 5-HT in microdissected raphe of experimentally infected laboratory-reared killifish, as functions of parasite density (number of parasites per g host body mass). Data were analysed using linear mixed-effects models. Best-fit trend lines are second order polynomial for both A and B, with R^2^ being 0.75 and 0.63, respectively, *n*=10.

As predicted, serotonergic activity declined with parasite density (LMM: *F*_(1,4)_=8.18, *P*=0.046, R^2^m=0.50, R^2^c=0.52; Fig. 1A). The change in this ratio was driven by declining 5-HIAA concentration with increasing parasite densities (LMM: *F*_(1,4)_=18.32, *P*=0.013, R^2^m=0.67, R^2^c=0.67; Fig. 1B). Conversely, 5-HT did not change systematically with parasite density (LMM: *F*_(1,4)_=0.38, *P*=0.57, R^2^m=0.04, R^2^c=0.04; Fig. 1C). The finding that [5-HIAA] is changing in response to parasite densities while [5-HT] is not suggests that parasites are modifying neurotransmission and not 5-HT availability for release.

None of the 5-HT parameters were significantly correlated with parasite intensity (total number of parasites in host) (LMM: [5-HIAA/5-HT] *F*_(1,3)_=3.03, *P*=0.180, R^2^m=0.89, R^2^c=0.94; [5-HIAA] *F*_(1,3)_=0.07, *P*=0.813, R^2^m=0.49, R^2^c=0.60; [5-HT] *F*_(1,3)_=1.88, *P*=0.263, R^2^m=0.22, R^2^c=0.22).

**Fig. 2.**
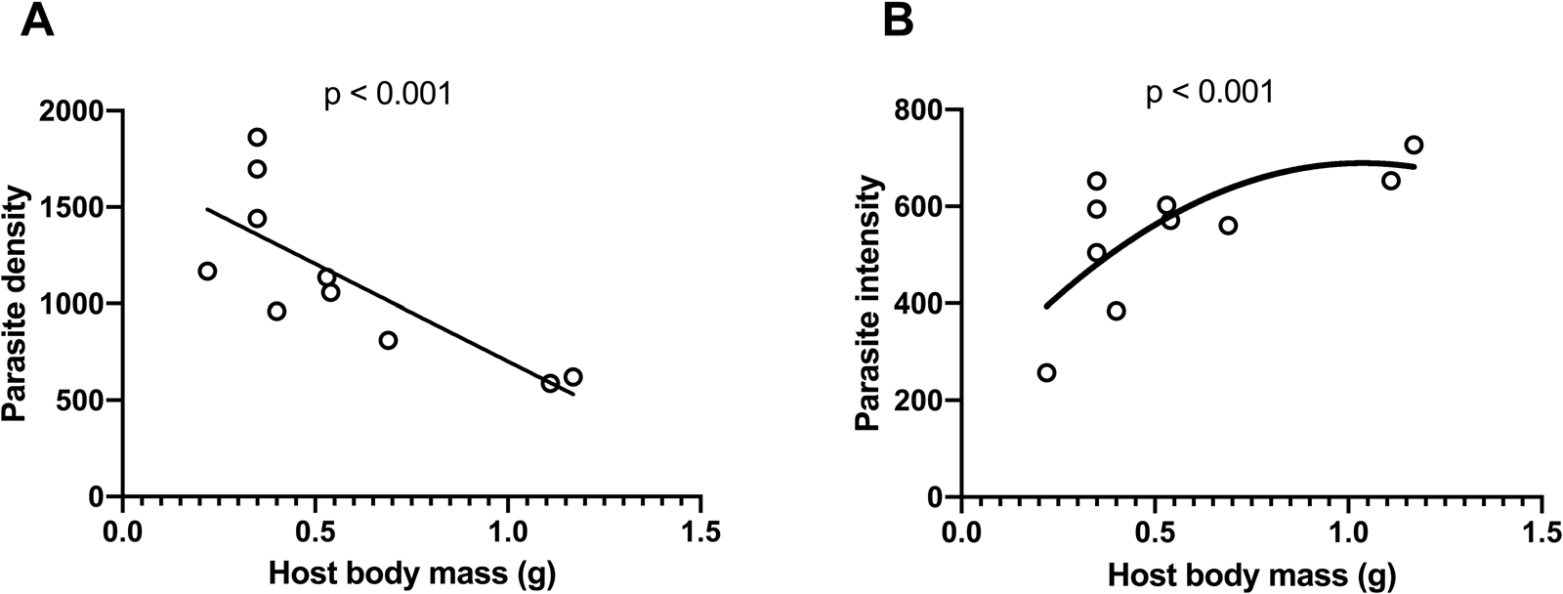
**Density and intensity of *E.californiensis* in California killifish (*F.parvipinnis*) and their relationship to host body mass.** (A) Parasite density (number of parasites per g host body mass) decreased in relation to host body mass and (B) parasite intensity (number of parasites in a host) increased in relation to host body mass in laboratory-reared killifish experimentally infected with *E. californiensis.* Data were analysed using generalized linear models. Best-fit trend line was linear in A and second-degree polynomial in B, with R^2^ being 0.60 and 0.50, respectively.

**Fig. 3.**
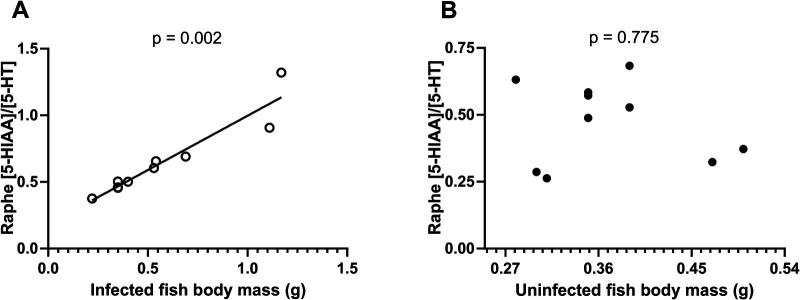
**The relationship between serotonergic activity and body mass in California killifish (*F.parvipinnis*) depends on *E.californiensis* infection.** Serotonergic activity ([5-HIAA]/[5-HT]) as function of host body mass (g) in (A) experimentally infected (open circles) fish from the KF Marsh Reserve in San Diego, CA, USA and (B) uninfected (filled circles) laboratory-reared killifish from SE in Cardiff, CA, USA. Data were analysed using linear mixed-effects models. The best-fit line for A was linear, with R^2^ being 0.90.

In infected fish, parasite density declined (LMM: χ^2^=30.29, *P*<0.001, R^2^m=0.45, R^2^c=0.89, Fig. 2A) whereas parasite intensity increased (GLMM: χ^2^=236.83.70, *P*<0.001, R^2^m=0.64, R^2^c=0.98, Fig. 2B) with host body mass. In other words, larger fish harboured more parasites in absolute numbers, but fewer parasites per g of tissue. As a consequence, for the infected fish, serotonergic activity increased with host body mass (LMM: *F*_(1,3)_=126.15, *P*=0.002, R^2^m=0.89, R^2^c=0.94; Fig. 3A). In contrast, uninfected fish exhibited no significant relationship between serotonergic activity and body mass (LMM: *F*_(1,4)_=0.09, *P*=0.775, R^2^m=0.00, R^2^c=0.80; Fig. 3B).

## DISCUSSION

In this study, we investigated post-stress serotonergic activity California killifish exposed to repeated experimental infections with the brain-encysting trematode *E. californiensis*. The results indicate that at ecologically relevant parasite loads, raphe serotonergic activity decreases with increasing parasite densities. Moreover, we found that density-correlated changes in 5-HT signalling were driven by concentrations of the 5-HT catabolite 5-HIAA, and not by 5-HT concentrations*,* which indicates that alterations in 5-HT neurochemistry are due to changes in 5-HT transmission and not available transmitters stores. In contrast, parasite intensity was not related to any aspect of 5-HT signalling. This indicates that the number of parasites per unit body mass (i.e. parasite density) rather than the total number of parasites (i.e. parasite intensity) determines the strength with which parasites influence 5-HT signalling. The importance of distinguishing parasite density and intensity was further highlighted by finding that these two parameters exhibited opposing relationships with host body mass, with density declining and intensity increasing in larger individuals. This relationship likely led to a rather uncommon observation, namely that in infected fish larger individuals had a higher serotonergic activity, a relationship that was not observed in uninfected fish. Typically, one would expect reduced 5-HT activity in larger individuals due to social stress (e.g. Winberg and Nilsson, 1993; Cubitt et al., 2008), or no systematic size dependent variation in cases were strong social hierarchies are absent. Associations between social rank and parasite load are not uncommon (Halvorsen 1986; Bartoli et al., 2000, and see review by Habig et al., 2018). This means that existing controversies regarding social rank and neuroendocrine function (see e.g. Sapolsky and Ray, 1989; Creel, 2001; Gesquiere et al., 2011; Winberg et al., 2016) could well be re-examined for possible roles of parasites and pathogens.

We observed that serotonergic activity decreased with increasing parasite density following a non-linear, negatively saturating curve. This implies the existence of a minimum threshold after which further decrease in serotonergic activity is no longer possible or beneficial for either the parasite or the host. After all, the serotonergic system has many vital roles (Chaouloff, 1993; Winberg and Nilsson, 1993; Summers et al., 2005; Summers and Winberg, 2006; Duffy-Whritenour and Zelikoff, 2008; Puglisi-Allegra and Andolina, 2015; Prasad et al., 2015; Andrews et al., 2015; Swallow et al., 2016; Backström and Winberg, 2017) so a complete disruption in serotonergic signalling could compromise both host survival and parasite transmission. The existence of such a threshold (or diminishing returns) would fit in with the hypothesis that *E*. *californiensis* benefit from crowding on killifish brains (Weinersmith et al., 2014). If parasite alterations of host behaviour are costly (e.g. costs related to production or secretion of neurologically active chemicals), then parasites under high density may be able to share the costs (Poulin, 1994; Brown, 1999). If such cost sharing is possible, then the parasites can invest saved resources into other fitness-influencing traits. Consistent with this idea, Weinersmith et al. (2014) documented that individual *E. californiensis* metacercariae were slightly larger when found in high densities. If manipulating serotonergic activity represents a costly form of behaviour alteration, the observed negatively saturating curve may underlie the cost sharing and benefits of *E. californiensis* in high-density infections.

Interestingly, the relationship between parasite density and serotonergic activity that we observed is strikingly similar to the pattern observed by Shaw et al. (2009), even though the parasite densities in that study were orders of magnitude lower than in ours. The consistent negatively saturating pattern implies that whether an absolute threshold for density dependent effects is reached, there may generally be a sub-additive effect of parasite density on host physiology.

Although we performed experimental infections, we cannot formally establish a causal relationship between parasite infection and altered serotonergic activity. Given the established links between 5-HT and immune function in fish as well as mammals (Khan and Deschaux, 1997; Shajib and Khan, 2015), inherent differences in serotonergic activity could make some individuals more susceptible to infection rather than parasites directly changing serotonergic activity. In such a scenario, however, we would expect to see strong associations between serotonergic activity and total numbers of parasites residing in the host. Such a relationship was not evident in our data. While it is possible that changes in serotonergic activity in response to parasite density are driven by the host to compensate for infection, the observed highly precise relationship between 5-HT activity and parasite density is consistent with direct manipulative effects of parasites on central nervous system function. Indeed, theory and empirical evidence suggest that parasite manipulation of highly integrated signal systems (such as 5-HT and other monoamines) involved in multiple physiological and behavioural traits would be an effective strategy to achieve adaptive phenotype manipulation by the parasites (Prandovszky et al., 2011; Lafferty and Shaw, 2013; Perrot-Minnot et al., 2014).

Our data further indicate that the observed association between parasite density and post-stress serotonergic activity is driven by [5-HIAA]. Because 5-HIAA is formed primarily after 5-HT release and reuptake, this indicates an inhibitory effect of parasites on neurotransmission rather than on available 5-HT stores. Notably, these effects are observed in microdissected samples of the raphe region, which contains the major serotonergic cell bodies found in the brain (Lillesaar, 2011). Hence, altered serotonergic activity in raphe brain region can have cascading effects on serotonergic signalling throughout all brain regions.

Infected and uninfected fish originated from different locations and we can therefore not rule out population differences for this particular result. However, due to the short geographical distance and expected connectivity between the two locations, we hypothesize that infection status is a major factor distinguishing these test populations, rather than, for example, differences in social dynamics. Theoretically, strong social hierarchies may have developed in infected but not uninfected fish, but if that were the case, the direction of effect would likely be the opposite of what was observed (i.e. higher serotonergic activity/responsiveness in smaller rather than larger individuals; Winberg and Nilsson, 1993; Øverli et al., 1999; Vindas et al., 2016). Hence, this result suggests that the positive association between serotonergic activity and host body mass was driven by the high densities of *E. californiensis* suppressing serotonergic activity in smaller fish. Hence, regarding studies in wild populations potentially infected by multiple species of parasites, neuroendocrine correlates with body size and associated life history outcomes should be evaluated in light of parasitism (particularly parasite density), which may influence or even reverse observed patterns.

### Summary and conclusions

We found that post-stress raphe serotonergic activity decreased with increasing brain parasite density, but not intensity, in California killifish experimentally infected with *E. californiensis* metacercariae. Notably, decreased 5-HT has previously been associated with increased parasite density in killifish (Shaw et al., 2009), but only at parasite loads well beneath those observed in most wild killifish. Our results show that serotonergic activity is also supressed at high, ecologically relevant infection intensities, which helps us understand the nature of this classic host-parasite system. Further, our results also clarify that it is the number of parasites per unit body mass (i.e. parasite density) rather than total number of parasites per host (intensity) that determines the strength by which parasites influence 5-HT signalling. This finding is also consistent with a previous study that compared effects of parasite density versus intensity on cortisol release rates in killifish (Weinersmith et al., 2016). Consequently, when studying effects of *E. californiensis* infection in killifish, parasite density is probably the most relevant measurement of parasitism. In addition, we show that the association between parasitism and serotonergic activity likely reflects a parasite-density dependent effect on 5-HT neurotransmission rather than availability of the neurotransmitter. This outcome may reflect that *E. californiensis* metacercariae directly suppress raphe 5-HT neurotransmission. If *E. californiensis* and/or other parasites are capable of actively influencing brain serotonergic activity, parasite infections could have far-reaching effects beyond the neurophysiological parameters investigated here. Future studies in this system should address potential effects of parasite infections on physiological processes (e.g. monoaminergic activity, reproductive and stress neuroendocrine axes) and behaviours (e.g. locomotor behaviour, activity and aggression) known to be modulated by 5-HT. Lastly, our analyses revealed a positive relationship between body size and post-stress serotonergic activity that was only present in infected fish and was driven by smaller fish harbouring higher parasite numbers per unit body mass. A main implication of this observation is that parasites may steer observed relationships between body mass and central neuroendocrine processes.

## MATERIALS AND METHODS

### Experimental animals

This work was approved by the Institutional Animal Care and Use Committee at the University of California, San Diego, following the laws and regulations controlling experiments and procedures on live animals in the United States. The laboratory-reared fish used in this study are a subset of fish from a larger, multifaceted project. Using a two-pole seine, California killifish were captured between July to September 2016 from two populations naturally infected with *E. californiensis*, Kendall-Frost (KF) Marsh Reserve in San Diego, CA, USA and San Elijo (SE) in Cardiff, CA, USA. Eggs and milt were mixed together in a petri dish containing a volume of seawater just large enough to cover the eggs. Fertilized eggs were brought back to Scripps Institution of Oceanography (SIO), and placed in round fingerbowls (diameter: 100 mm) containing 200 ml of aerated and filtered seawater at a density of 250 eggs per l. The last group of eggs received filtered seawater mixed with Methylene Blue, 0.0003%, until day 17–19 post-fertilization to increase egg survival. Eggs were kept on a light:dark cycle tracking the local day length. Dead or unfertilized eggs were removed daily. A complete water change was done every other day until hatching at approximately 21 days post-fertilization. Hatched fry were transferred to 37.8 l aquaria and maintained in groups of 20. Each tank got a set of 20 fish that were hatched at various times (fish used here were hatched at five different occasions with no more than 2 months separating the first and last hatch), resulting in 11 tanks in total. During the first 12 weeks of life, fish were fed live brine shrimps once daily. After 12 weeks post-hatch, fish were transitioned to a more varied diet composed of blood worms, aquaculture feed (Skretting Protec Starter) and mashed peas.

### Experimental infections

Many studies that use experimental infections achieve infections by exposing hosts to one large dose of parasites (e.g. Shaw et al., 2009). However, in nature, killifish achieve high parasite intensities through the slow accumulation of parasites throughout their lifetimes. We used a long-term, repeated infection method to more accurately mimic natural exposure patterns (more fully described in Helland-Riise et al., 2020). In short, experimental infections were carried out continuously from when fish were approximately 8 weeks post-hatch, throughout the life of the fish (approximately a 9-month infection period, September 2016 – June 2017). Cercariae (a free-swimming stage of the parasite infectious to killifish) in this system emerge from snails when inundated with seawater, with cercariae ‘shedding’ at the highest rates during the warm summer months (Fingerut et al., 2003). In our experiments, fish were typically infected twice weekly. However, on nine occasions during the winter months (when temperatures were cooler and natural shedding rates would also be lower), we were unable to complete one of the weekly infections due to insufficient shedding. In total, fish were subjected to 61 infection events. California horn snails (*Cerithideopsis californica*) originating from KF were used to obtain *E. californiensis* cercariae (cercariae identified following Hechinger, 2019). Snails were identified as harbouring exclusively *E. californiensis* by shedding them individually in compartment boxes on three separate occasions over a 2-week period and visually inspecting the cercariae under a microscope, prior to using them in experimental infections. We maintained the snails in mudflat mesocosms operating under an artificial tidal regime mimicking the local tidal cycle. For each experimental infection, approximately 120 horn snails previously identified as harbouring *E. californiensis* were removed from the mesocosms and placed in a humid environment for a minimum of 24 h before shedding. 2–4 h before an infection event, groups of seven to nine snails were placed in finger bowls (10 cm internal diameter) containing filtered seawater heated to 27°C and placed under a fluorescent light. Parasite identity was again confirmed visually using a microscope and the number of *E. californiensis* cercariae shed was recorded.

Fish received either a parasite treatment (cercariae+seawater) or sham treatment (seawater only). Cercarial exposure per fish increased continuously over the course of the experiment as the fish grew. For the parasite infection group, the number of parasites added to the tank ranged from 1–124 cercariae per fish on average per exposure throughout the experiment. During the first 12 weeks of infections, cercariae were individually counted and placed in scintillation vials (24 mm diameter×61 mm height; volume 20 ml), which were topped off with warm (28°C), filtered seawater. Once infection exposures exceeded 12 cercariae per fish, parasites were allocated by volume of parasite laden seawater. The number of parasites in all finger bowls were estimated using sub-sampling counting methods, then were pooled and aliquoted into Qorpak jars (56 mm diameter×70 mm height; volume 120 ml). Each tank had a designated jar, which was slowly lowered to the tank bottom using monofilament-fishing line. Fish receiving a control treatment (i.e. uninfected) were sham treated with jars containing cercaria-free seawater that was otherwise treated the same way as cercaria-laden water. Jars were removed from tanks 18–24 h after infection. Due to high mortality of KF fish (ended up with two tanks), the control fish used in this experiment all originated from the SE population (ended up with three tanks). However, fish populations at KF and SE are likely quite similar. The two wetlands are only 25 km apart and probably frequented by the same birds (and thus exposed to the same parasite population). Moreover, due to the short geographical distance, we expect some connectivity and gene flow between these populations (Bernardi and Talley, 2000). Moreover, although KF and SE fish originated from different populations, they were reared in the same laboratory under identical conditions.

### Stress tests and sampling

Experimental fish were approximately 10 months old when they were sampled. As previously stated, the purpose of this study was to determine effects of parasite infection on post-stress serotonergic activity levels. However, this study was part of a larger, multifaceted project where an additional aim was to characterize killifish behaviour during different types of stress (e.g. behavioural responses to novel environment, simulated predator attack and confinement, data not included here). The stress exposure for the current paper therefore consisted of a series of sequential events: individual capture, transport, novel environment, a simulated predator attack and finally confinement stress. Fish were captured from their home tank using a net, then transported in a small transparent plastic container (89 mm×95 mm; diameter×height) that was placed on crushed ice to help maintain the seawater temperature. Transport took no more than 3 min from capture to transfer to the novel environment testing arena (135 mm×215 mm×135 mm; width×depth×height; filled with 1.3 l aerated seawater), where fish were left undisturbed for 30 min. A steel ball (1 cm in diameter) was then dropped into the arena (i.e. simulated aerial predator attack) before fish were left undisturbed for an additional 30 min. Lastly, fish were subjected to confinement stress in a small, circular, transparent plastic container (63 mm×67 mm; diameter×height) filled with 0.016 l seawater. Three such containers (each housing one fish) were placed in a square box (270 mm×390 mm×168 mm) filled with aerated seawater. The circular containers had four small holes at the bottom to allow aeration of the fish holding water. The three fish tested in this communal water source originated from the same holding tank, and are hereafter referred to as a ‘batch’. After 30 min, experimental fish were placed in a lethal dose (250 mg l^−1^) of buffered tricaine methanesulfonate (MS-222) solution until no opercular movements could be observed. Individual body mass was recorded, before the head was removed with a scalpel and placed on dry ice before storage at −80°C. The fishes' gonads were not developed, and we could not determine their sex.

### Tissue processing, microdissection and parasite quantification

Frozen heads were mounted in Tissue-Tek^®^ (Sakura Finetek) and placed on dry ice within 30 s. Embedded tissue was sliced with a cryostat (Leica, CM 3050) at −20°C in serial 60 µm slices. The slices were thaw mounted onto microscope slides (Menzel-Gläser, Thermo Fisher Scientific), then refrozen and stored at −80°C. Microscope slides were placed on a cold stage (−14°C) underneath a dissecting microscope and the raphe nuclei within the brain stem area were identified using a stereotaxic atlas (Wulliman et al., 1996) and microdissected using a modified 26 G needle. This area was chosen since 5-HT neurons in the raphe synthesize the majority of brain 5-HT and innervate all other brain areas (Lillesaar, 2011). Microdissected tissue was immediately ejected into 71 µl sodium acetate buffer (pH=5).

**Fig. 4.**
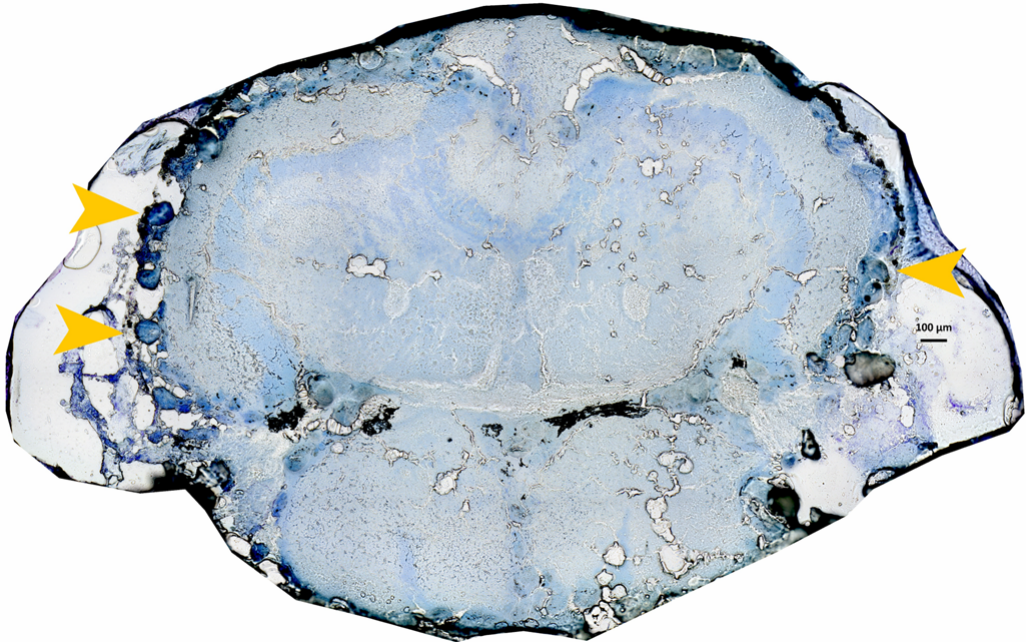
**Cresyl Violet Nissl-stained brain section showing *E.californiensis* parasite cysts in the California killifish (*F.parvipinnis*).** Midbrain (diencephalon/mesencephalon) killifish section in light blue where orange arrows indicate parasite cysts.

To facilitate parasite quantification, slides were thawed after microdissections at room temperature for 1 h and the tissue was post-fixed by immersion in 70% EtOH for 2 min. Immediately after, the tissue was stained by submerging slides in: 1) a Cresyl Violet solution [containing 2 g of Cresyl Violet (Sigma-Aldrich, Darmstadt, Germany), 100 ml of RNase free water and 100 ml of 100% EtOH] for 45 s, 2. 70% EtOH for 10 s, and 3) 100% EtOH for 10 s. Following this, slides were air-dried, mounted with a cover glass and sealed in place using a film-forming polymer (Isadora, Malmö, Sweden). The slides were then stored at room temperature. Thereafter, high-resolution images (10× magnification) of the stained sections of the infected fish were acquired using an automated slide scanner system (Axio Scan Z1, Carl Zeiss Microscopy, Munich, Germany). Images were analysed visually, and parasites were quantified by counting metacercariae on the diencephalon/mesencephalon only, because the number of parasites found in this area is highly correlated to total parasite numbers (Helland-Riise et al., 2020). Note that microdissections did not dislocate metacercariae, as the parasites are located on the brain surface (see Fig. 4 for a representative picture).

### Monoamine analysis

Samples containing microdissected tissue were thawed on ice and centrifuged at 15,000 ***g*** at 4°C for 5 min. Supernatant was removed and used for high-pressure liquid chromatography (HPLC) analysis, while the pellet was refrozen at −80°C for protein analysis by a Bradford protein assay as described by Vindas et al. (2014). The HPLC system consisted of a solvent-delivery system (Shimadzu, LC-10AD), an auto injector (Midas, Holland Spark, The Netherlands), a reverse phase column (4.6×100 mm, Hichrom, C18, 3.5 mm) and an ESA Coulochem ΙΙ detector (ESA, Bedford, MA, USA) with two electrodes at −40 mV and +320 mV. A conditioning electrode (potential of +40 mV) was used to oxidize any contaminants before analysis. The mobile phase consisted of 86.25 mM sodium phosphate, 1.4 mM sodium octyl sulphate and 12.26 µM EDTA in deionized water (resistance 18.2 MW) containing 7% acetonitrile brought to pH 3.1 with phosphoric acid. Brain concentrations of 5-HT and its catabolite 5-HIAA were quantified by comparing them with standard solutions of known concentrations using HPLC software (CSW, DataApex Ltd, Czech Republic).

### Statistics

All statistical analyses were conducted in the R Statistical Environment [v3.2.4, (R Development Core Team, 2018)], using the R base package and the packages ‘lme4’, ‘nlme’ and ‘MuMin’ (Pinheiro et al., 2017; Bumham and Anderson, 2002). For all models, to ensure meeting assumptions concerning normality and homoscedasticity, residual and quantile-quantile plots were inspected visually (Pinheiro et al., 2001; Zuur et al., 2010). Using linear mixed-effects models (LMM), serotonergic activity (as measured using the ratio between 5-HIAA/5-HT) and the concentrations of 5-HT and 5-HIAA were analysed with infection density as a continuous explanatory variable, and batch nested within tank (with tank number referring to the tank in which the fish were held long term during development) as a random effect. In addition, LMMs were used to assess the role of body mass and infection intensity as continuous explanatory variables in all three measures of 5-HT parameters (serotonergic activity, 5-HT and 5-HIAA) in infected fish (*n*=10), with batch (five batches in total) nested within tank (two tanks in total) as a random effect. Serotonergic activity was analysed for uninfected fish (*n*=10) in a separate LMM (as they originated from a different population), with body mass as a continuous explanatory variable and batch nested within tank as a random effect. For all LMM models, the marginal and conditional R^2^ was calculated (hereafter denoted by R^2^m and R^2^c, respectively) to quantify the proportion of variance explained by the fixed factors only and the fixed and random factors, respectively. To meet the assumptions of the LMM models, all dependent variables were log-transformed, except the serotonergic activity of uninfected fish, which did not require transformation. A generalized linear mixed-effects model (GLMM) permitted assessing the role of body mass in infection intensity (run using a Poisson distribution), and an LMM was used to investigate links with infection density (log-transformed to meet model assumptions), with both models including a random effect for tank of origin as fish were group-infected in their home tank. Batch (from stress protocol) was not necessary to include in these models as fish mass, infection intensity, or infection density could not possibly be influenced by that factor. JMP pro 14 was used to determine the best-fit trend line. The best trend line was defined as having the highest R^2^-value and the lowest root mean square error.
